# Hippocampal gamma predicts associative memory performance as measured by acute and chronic intracranial EEG

**DOI:** 10.1038/s41598-018-37561-z

**Published:** 2019-01-24

**Authors:** Simon Henin, Anita Shankar, Nicholas Hasulak, Daniel Friedman, Patricia Dugan, Lucia Melloni, Adeen Flinker, Cansu Sarac, May Fang, Werner Doyle, Thomas Tcheng, Orrin Devinsky, Lila Davachi, Anli Liu

**Affiliations:** 1 New York University Comprehensive Epilepsy Center, 223 East 34th Street, New York, NY 10016 USA; 2 Department of Neurology, New York University School of Medicine, 240 East 38th Street, 20th Floor, New York, NY 10016 USA; 3 NeuroPace, Inc., 455 N. Bernardo Avenue, Mountain View, CA 94043 USA; 4 Department of Neuroscience, Max Planck Institute for Empirical Aesthetics, Gruneburgweg 14, 60322 Frankfurt am Main, Germany; 5 Department of Psychology, New York University, 6 Washington Place, New York, NY 10003 USA; 6 Department of Neurosurgery, New York University School of Medicine, 530 1st Avenue, Suite 7W, New York, NY 10016 USA; 7 Columbia University, Department of Psychology, 1190 Amsterdam Ave #406, New York, NY 10027 USA

## Abstract

Direct recordings from the human brain have historically involved epilepsy patients undergoing invasive electroencephalography (iEEG) for surgery. However, these measurements are temporally limited and affected by clinical variables. The RNS System (NeuroPace, Inc.) is a chronic, closed-loop electrographic seizure detection and stimulation system. When adapted by investigators for research, it facilitates cognitive testing in a controlled ambulatory setting, with measurements collected over months to years. We utilized an associative learning paradigm in 5 patients with traditional iEEG and 3 patients with chronic iEEG, and found increased hippocampal gamma (60–100 Hz) sustained at 1.3–1.5 seconds during encoding in successful versus failed trials in surgical patients, with similar results in our RNS System patients (1.4–1.6 seconds). Our findings replicate other studies demonstrating that sustained hippocampal gamma supports encoding. Importantly, we have validated the RNS System to make sensitive measurements of hippocampal dynamics during cognitive tasks in a chronic ambulatory research setting.

## Introduction

The hippocampus is critical to forming novel associations between previously unrelated items^1–3^, binding information across time and space^4^. Prior studies investigating the neural basis for successful episodic learning have described gamma (40–100 Hz) increases with simultaneous decreases in theta (5–8 Hz) in the hippocampus and widespread cortical regions during successful encoding, distinguishing items that are later successfully remembered versus forgotten, termed the subsequent memory effect^5–7^.

Previous studies have utilized episodic memory tasks^6–8^ measured in epilepsy patients undergoing invasive electroencephalography for resective surgery. Traditional iEEG, involving a combination of subdural grids, strips, and depth electrodes, is considered the gold standard for fine-grained dissection of the spatiotemporal dynamics of cognitive processing. Compared to noninvasive methods, such as scalp EEG or functional magnetic resonance imaging (fMRI), iEEG recordings offer an excellent signal-to-noise ratio and direct measurements of population-level neuronal responses at millisecond resolution. Yet, traditional iEEG is limited to memory investigations studied across short timescales, as well as by subject-related limitations of pain, seizures, and distraction inherent to the clinical scenario. In particular, some of the experimental constraints of traditional surgical iEEG research include: (1) Suboptimal patient participation due to clinical factors including seizures, pain, medications, fatigue, and disrupted sleep-wake cycles, (2) Lack of control over the hospital environment which may cause distraction during cognitive testing^9^, (3) 60 Hz line noise generated from nearby equipment (e.g. hospital bed, compression stockings, and monitoring equipment) and frequent interictal epileptiform discharges, resulting in significant data loss, and (4) limited duration of recording to 3–16 days due to infection risk and degradation of signal quality.

The RNS System (NeuroPace, Inc.) is an FDA-approved therapy to treat medically refractory focal seizures in adults^10,11^. When adapted for research purposes, this system can potentially address some of the limitations of traditional iEEG during cognitive investigations. As a therapeutic device, the RNS System detects abnormal patterns of electrical activity, then delivers brief pulses of electrical stimulation in a closed loop manner. The system includes two four-contact leads placed on or in seizure foci, which record and store changes in local-field potentials (LFP) with millisecond precision. While the RNS System is a clinical therapy, the availability of chronic electrocorticography permits the investigation of brain dynamics in the ambulatory setting^12^.

**Figure 1:**
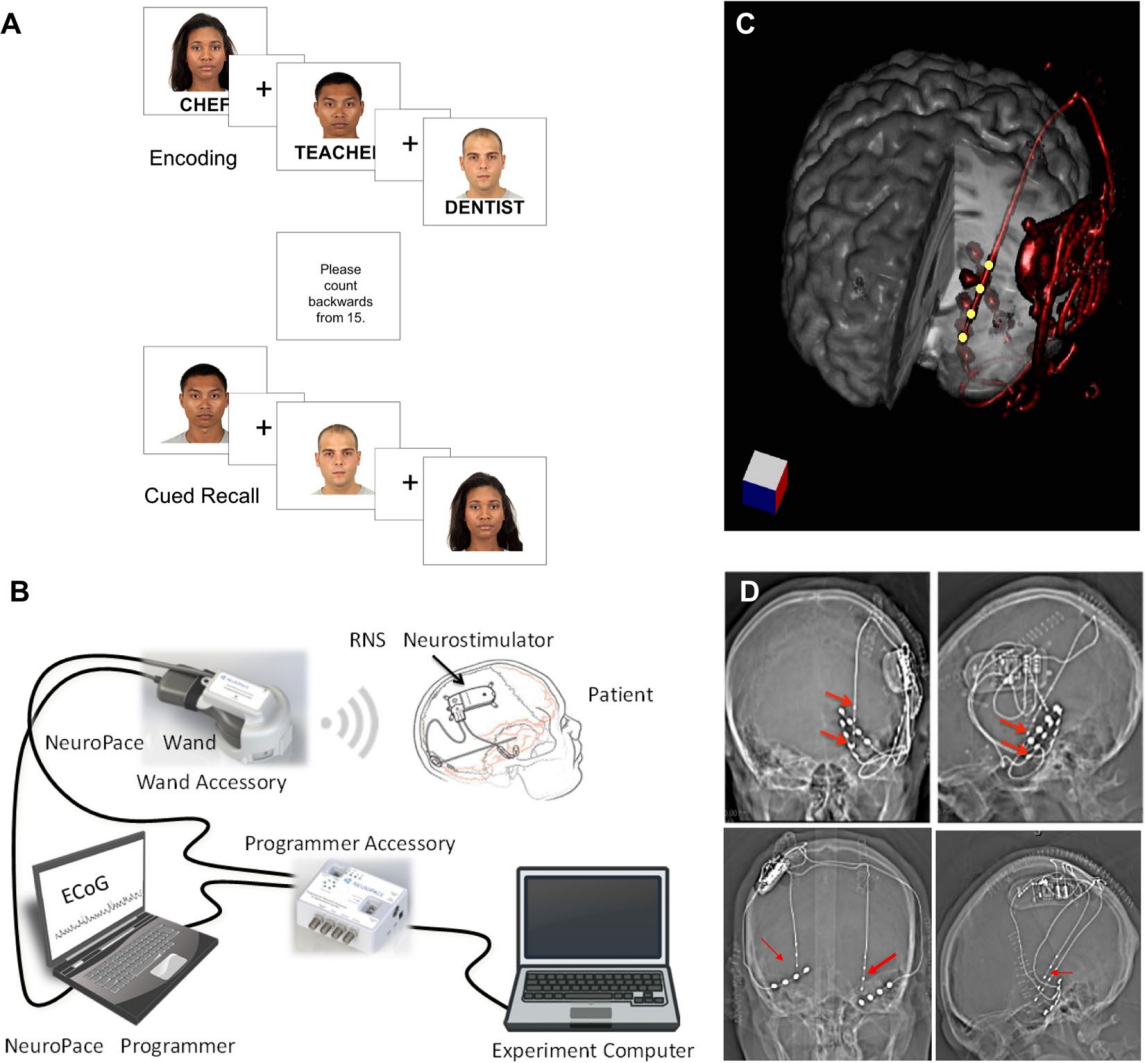
RNS System patients and experimental system. (**A**) During the face-profession task, patients were shown pairs of novel faces and common professions and asked to form an association. Following a distraction task, patients were cued with the previously seen faces and asked to freely recall the associated professions. (**B**) The clinical RNS System and Research Accessories (RAs), including Wand Accessory (WA) and Programmer Accessory (PA). (**C**) 3-dimensional reconstruction of neurostimulator and lead placement (red). Yellow dots are hippocampal depth lead electrodes, which can record and stimulate (although stimulation was turned off during the experiment). (**D**) Top Row. Head CT of Patient R1 with neurostimulator and left hippocampal depth and mesial temporal subdural lead electrodes (red arrows), coronal (left) and sagittal (right) views. Additional leads present but not connected to neurostimulator, include: left basal temporal neocortical and lateral temporal neocortex. Bottom Row. Head CT of Patient R2 with neurostimulator and bilateral hippocampal depth lead electrodes, with coronal and sagittal views. Additional leads present but not connected to neurostimulator are located in the bilateral basal temporal regions. Patient R3 had similar bilateral hippocampal depth electrode placements (not shown). NeuroPace and RNS are registered trademarks of NeuroPace, Inc. Faces in Panel A are reused from the Chicago Face Database (CFD) under the Creative Commons Attribution 4.0 open access license^15^. @Neuropace, Inc image from Panel B is reused with permission from NeuroPace, Inc.

In this study, we aimed to replicate previous work on hippocampal physiology predictive of successful encoding using an associative memory paradigm in a traditional surgical iEEG group and extend these investigations to a chronic ambulatory iEEG (RNS System, NeuroPace, Inc.) population. We selected a face-profession association task, a memory task which has produced robust fMRI activation in bilateral hippocampi^13^, and is similar to a face-name association task which has been demonstrated to be sensitive to memory decline in a clinical population^14^ (Fig. 1A).

To measure the hippocampal physiology of associative learning, we performed this face-profession paradigm in five patients with conventional iEEG. We performed the same paradigm in 3 RNS System patients, adapting the device to permit task synchronization to the RNS System iEEG without modifying the clinical system **(**Fig. 1B**)**.

The associative memory paradigm was administered in two phases. During the encoding phase, patients were shown a series of color pictures of faces paired with professions and were asked to read aloud the profession to ensure attention. A brief distraction task was then administered. During the cued recall phase, faces were presented in a randomized order and participants were asked to name the paired profession (see Fig. 1A). The task was administered to five (5) surgical patients implanted with hippocampal depth leads and to three patients previously implanted with the RNS System (Fig. 1C,D), during testing sessions spanning several months. In this IRB approved study, real-time iEEG recording from the RNS Neurostimulator and integration with a testing platform was enabled by IRB-approved Research Accessories (RAs), permitting task-based study design and analysis (Fig. 1B). These adults with drug-resistant focal onset epilepsy were implanted with the RNS System in accordance with FDA-approved indication for use and for reasons completely independent of this research study. Patients who were selected for participation had a cranially implanted brain-responsive programmable neurostimulator connected to hippocampal-placed depth leads and were capable of consent. We describe the clinical characteristics of the eight (8) patients (3 RNS System, 5 surgical) in Table 1 and in the Supplementary Material section. We later analyzed hippocampal iEEG during encoding, comparing trials which were later remembered versus forgotten.

**Table 1:** Subject characteristics and test performance.

Subj ID	Age	Sex	Hand	Lead/Electrode Coverage	FSIQ	Session Number	Date	# of trials	% correct	Average Block Size	# Trials Rejected
R1	21	M	R	L Hipp depth; L basal temp strip	108	1 of 3	May 2017	78	65.4	8.7	5
						2 of 3	October 2017	114	50.9	11.4	5
						3 of 3	January 2018	118	48.3	14.8	10
R2	45	M	R	L hipp depth; R hipp depth	84	1 of 1	April 2017	36	19.4	6.0	2
R3	32	F	R	R hipp depth	96	1 of 2	November 2017	118	53.4	6.9	4
						2 of 2	January 2018	94	47.3	7.8	3
S1	45	F	R	L and R hipp depths (2 + 2); L and R frontal, temporal, parietal, occipital strips (8 + 8)	99	1 of 1	January 2017	106	79.2	2.3	10
S2	18	M	R	L and R hipp depths (2 + 2); L and R frontal, temporal, parietal, and occipital strips (8 + 8)	91	1 of 1	July 2017	70	42.9	8.8	8
S3	37	M	R	L and R hipp and insular depths (3 + 3); L and R frontal, temporal, parietal, and occipital strips (5 + 5)	82	1 of 1	October 2017	94	40.4	4.5	62
S4	20	F	R	R hipp and insular depths (5); R hemisphere (2 × 64 contact grids; 6 frontal and temporal strips);	74	1 of 1	December 2017	119	47.1	4.1	7
S5	25	M	R	R hipp depths; R hemisphere	134	1 of 1	February 2018	92	46.7	9.2	7

## Methods

### Participants and Recruitment

This study was performed in epilepsy patients with previously implanted RNS Systems for treatment of refractory focal epilepsy. Research with RNS System patients was performed in accordance with protocol approved by the New York University School of Medicine Institutional Review Board (NYUMC), and with written patient consent. Subjects were eligible if they (1) were adults (18–70 years), (2) had the RNS System with at least one hippocampal depth lead, (3) had a FSIQ > 70, (4) were able to provide informed consent, and (5) were Native English speakers.

We also collected data from a group of patients undergoing surgical evaluation with intracranial EEG (iEEG) monitoring at NYUMC. The protocol was approved by the NYUMC Institutional Review Board and the Clinical Trials Registration number was NCT02263274 (www.clinicaltrials.gov). Subjects were eligible according to pre-established criteria, including: (1) age over 18 years old; (2) undergoing invasive monitoring for seizure localization for epilepsy surgery; and (3) ability to provide informed consent or have a legal guardian who could consent. Exclusion criteria included (1) significant cognitive impairment (IQ < 70) and (2) contraindication to MRI. All patients provided informed consent. Subjects were enrolled between January 2017 and February 2018. Table 1 lists subject characteristics.

### Face-profession Association Task

A computer-based face-profession task was used to test associative memory. A cued recall paradigm was chosen because it offers increased experimental control, especially regarding stimulus presentation and trigger alignment, compared to free recall. A bank of 240 high resolution color images of distinct human faces with neutral expression were taken from the Chicago Face Database^15^ (Fig. 1A). Each test set of faces were made up of an equal proportion of male and female White, Black, Hispanic, and Asian faces. Each face was randomly paired with an emotionally neutral, single-word profession between 4–10 letters in length, selected from the US Bureau for Labor Statistics database.

Given that performance varies greatly between subjects and to maximize statistical power, the task difficulty was adjusted depending on individual patient performance in an initial practice set. Between 2 and 20 paired stimuli were presented to each subject within each block (see Table 1). Each paired stimulus was presented for 5000 ms with a 1000 ms inter-stimulus interval. To ensure attention and sensory processing of test stimuli, patients were instructed to read the profession aloud and make a mental association.

Individual encoding blocks were followed by a brief distraction task to prevent rehearsal. Then, during the cued recall session, patients were shown the faces from the encoding session, in a pseudo-randomized order, and asked to verbally recall the associated profession. Responses were recorded and scored for offline analysis.

### RNS System and Research Platform

The RNS System is an FDA-approved medical device that provides brain-responsive (closed-loop) neurostimulation for patients with medically refractory epilepsy^10,11^. RNS System patients were chronically implanted with the RNS System for clinical reasons completely unrelated to research. Strategies for intracranial electrodes differed across patients with respect to lead type (subdural strip and/or depth) and implantation site. The lead type selection and implantation decisions were dependent on the clinical indication.

The RNS System includes two four-electrode leads, with 10 mm spacing (from electrode center to center), placed on or in the seizure foci. Each channel recorded LFPs in a fixed bipolar montage between adjacent electrodes. The electrocorticogram is filtered between 4–120 Hz (−3.5 dB at 4.5 and 100 Hz), and digitized at 250 Hz with 10-bit A/D resolution. When used for clinical reasons, short electrocorticographic (ECoG) recordings are stored by the neurostimulator during potential electrographic seizure activity, predetermined scheduling, or a magnet swipe. In a research setting, the RNS System’s live recording mode, using a wireless wand, is used to capture real-time ECoG recording.

**Figure 2:**
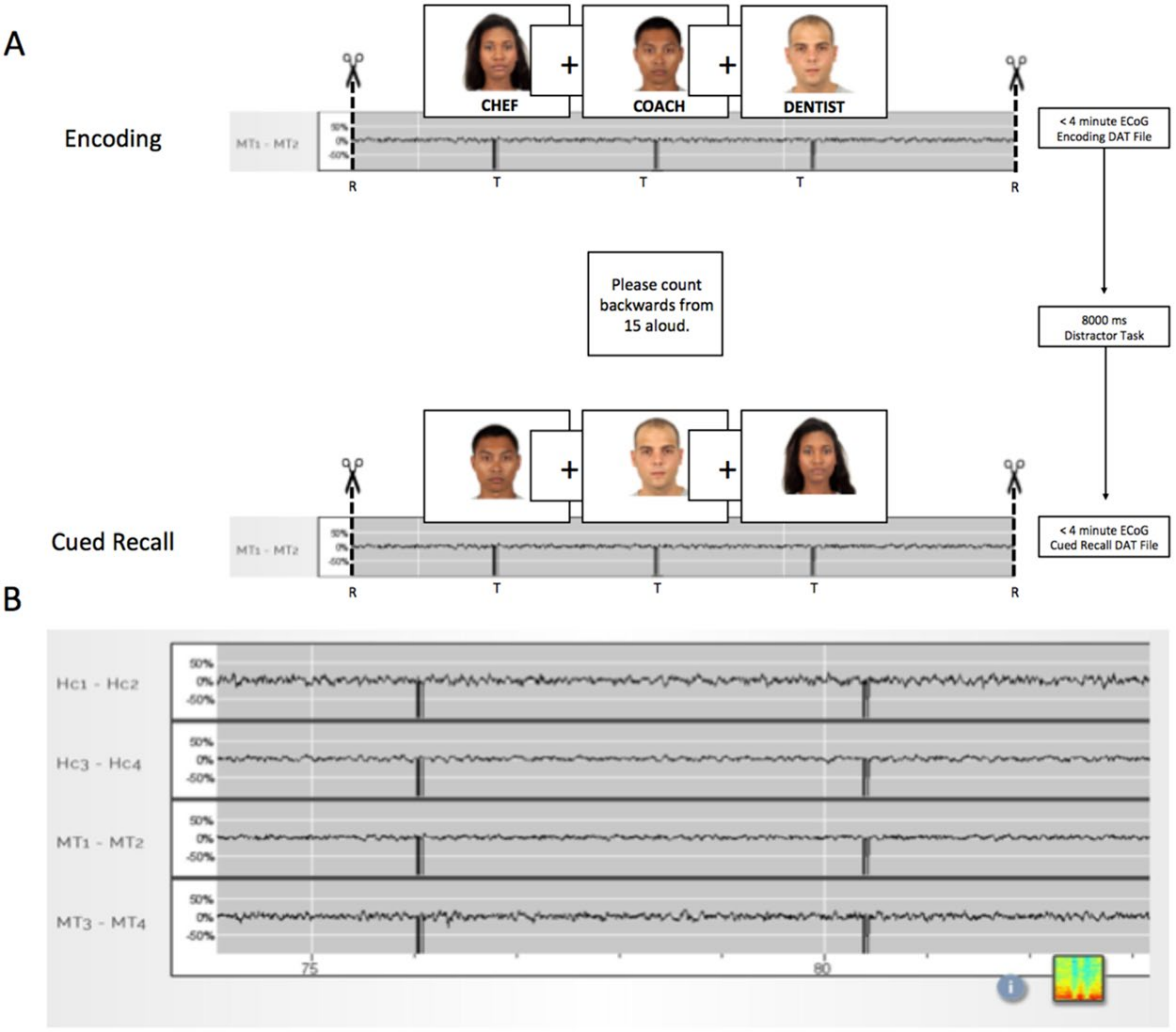
Coordination of task triggers to the RNS System iEEG (ECoG). (**A**) Example of RNS System iEEG recording during the Face-Profession Task. Trigger markers are seen as sudden signal dropouts (“telemetry artifacts”) in the recording and were delivered at the stimulus onset. As the RNS System records a maximum of 4 minutes of iEEG data at a time, each experimental block (either encoding or retrieval) was designed to be less than 4 minutes long. Triggers were sent via the programmer accessory (PA) to the wand accessory (WA), to mark the real-time ECoG with each stimulus onset. At the end of each experimental block, the task software commanded the PA to pause, store, and restart recording. This design allowed for continuous behavioral testing, task-locked ECoG recording, and avoidance of iEEG data loss. (**B**) Sample RNS System iEEG recording from 4 leads showing closeup of telemetry markers. Top 2 channels are recording from the hippocampal depth electrode. Bottom 2 channels are recording from the middle temporal gyrus. Faces in Panel A are reused from the Chicago Face Database (CFD) under the Creative Commons Attribution 4.0 open access license^15^.

To facilitate research utilizing the RNS System, several non-product tools (“Research Accessories”, RAs) were designed to enable external control of several basic RNS System functions and allow for synchronization with an experiment computer without modifying the FDA approved product. The RAs do not modify the RNS System, rather they use existing features of the clinical device while providing a computer-based method for integrating real-time markers into the ongoing recording stream (See Fig. 2), allowing for event-related neural response acquisition and analysis.

The task computer was connected to the Programmer Accessory (PA) via a universal serial bus (USB) communication link. Triggers were sent to the PA at the onset of each stimulus presentation. Because the RNS clinical system can only record iEEG segments for a maximum of 4 minutes duration, we designed each experimental block to be less than 4 minutes. Each block consisted of either the encoding or retrieval phase of the task, each presenting up to 20 Face-Profession paired stimuli per block, calibrated to the performance of each subject. Between each block, the task software commanded the PA to pause, store, and restart recording. This design allowed for continuous behavioral testing, task-locked ECoG recording, and avoidance of ECoG data loss (Fig. 2).

Responsive stimulation was suspended during task participation to avoid interference with endogenous neural rhythms during participation. An epilepsy physician (AL, PD, DF) monitored the RNS System iEEG (a.k.a ECoG) activity to ensure that no subclinical seizures occurred during testing, and to manage the patient in the event of a clinical seizure.

To permit sensitive detection of gamma band activity, the neurostimulator’s low-pass filter was adjusted from its default setting of 90 Hz to 120 Hz during testing and recording. Given that the RNS System iEEG is recorded in the bipolar montage between adjacent electrodes, we also analyzed the conventional EEG data using the bipolar montage. Bipolar recording is a fixed feature of the RNS System. Unlike conventional EEG or intracranial EEG, it is not possible to re-reference RNS System recordings in other montages, including the average montage. Given the technical constraints of the neurostimulator, and the desire to standardize analytic techniques between RNS system and surgical iEEG, the surgical iEEG was re-referenced to a bipolar montage for the primary analysis. Further signal processing details are given below.

The RAs include: (1) an accessory for the Programmer laptop (Programmer Accessory, PA) and (2) an accessory for the Wand (Wand Accessory, WA). The WA consists of a custom circuit board, an MSP-430 microcontroller (Texas Instruments), an electromagnet and a telemetry coil contained in a 3D printed case that encloses the Wand. The WA is controlled by commands received from the PA. The PA consists of an Arduino Due Microcontroller (“Arduino”) running custom software and a custom printed circuit board. The PA communicates with the experiment computer via a USB control interface. The Arduino is also connected to the Programmer laptop via USB and emulates a hardware mouse and keyboard to provide control inputs to the Programmer. The WA, while placed over the neurostimulator, allows live (real time) ECoG data to be viewed on the Programmer laptop in a continuous manner. ECoG data storage is normally limited to 4 minutes by the clinical system before it must be stored with several manual button clicks on the Programmer laptop.

The first function of the RAs automates storage of the ECoG data by executing a series of mouse movements and mouse clicks on the Programmer laptop graphical user interface (GUI). This is performed by stopping a currently running real time ECoG (clicking the “Stop” button), storing it to the Programmer’s hard disk (clicking the “Store” button), and then restarting the ECoG streaming (clicking the “Start” button). This configuration allows a single USB command from the experiment computer to control ECoG storage in a repeatable manner with precise timing that would not be possible if these tasks were performed manually without the RAs. This command introduces a minimal delay (~15 seconds) between recording sessions.

A second function of the RAs instructs the WA to insert a pattern of markers into the telemetry signal of the streaming ECoG data received from the neurostimulator by the Wand. This is equivalent to briefly moving the Wand away from the neurostimulator resulting in a telemetry dropout artifact. This distinctive pattern provides a means of reliably marking (“trigger marking”) the ECoG data within 2 ECoG samples (±4 ms) of the command. This marker is later used to provide synchronization of the time-series data between the experiment computer and the stored ECoG records.

### Post-surgical Intracranial EEG Recordings

Traditional intracranial EEG (iEEG) was recorded from implanted subdural platinum-iridium electrodes embedded in silastic sheets (2.3 mm diameter contacts, 10 mm center-center spacing, Ad-Tech Medical Instrument, Racine, WI) or depth electrodes (1.1 mm diameter, 5–10 mm center-center spacing). The decision to implant, placement of recording electrodes, and the duration of invasive monitoring were determined solely on clinical grounds and without reference to this study. Electrodes were arranged as grid arrays (8 × 8 contacts, 10 or 5 mm center-to-center spacing), linear strips (1 × 4 to 12 contacts), or depth electrodes (1 × 8 or 12 contacts), or some combination thereof. Subdural electrodes covered extensive portions of lateral and medial frontal, parietal, occipital, and temporal cortex of the left and/or right hemisphere.

Intracranial EEG is continuously streamed to a central monitoring station connected to a 40TB storage system at NYU Langone Health Hospital. A closed-circuit TV system complements the EEG recordings. Recordings at NYU are conducted in a 16-bed telemetry ward. Within 24 hours after surgical implantation of electrodes, patients underwent a post-operative brain MRI to confirm subdural electrode placement. Electrodes were localized and mapped onto the pre- and post-implant MRI using geometric models of the electrode strips/grids and the cortical surface^16^.

Recordings from grid, strip and depth electrode arrays were made using a NicoletOne C64 clinical amplifier (Natus Neurologics, Middleton, WI), bandpass filtered from 0.16–250 Hz and digitized at 512 Hz. Intracranial EEG signals were referenced to a two-contact subdural strip facing towards the skull near the craniotomy site. A similar 2-contact strip screwed to the skull was used for the instrument ground.

### Electrode Localization and Electrode Selection

Electrode localization was performed using automated processes and expert review. Pre-surgical and post-surgical T1-weighted MRIs were acquired for each patient, and the location of the electrode relative to the cortical surface was determined from coregistered MRIs following the procedure described in Yang *et al*.^16^. Co-registered, skull-stripped T1 images were nonlinearly registered to an MNI-152 template and electrode locations were then extracted in Montreal Neurological Institute (MNI) space (projected to the surface) using the co-registered image. A three-dimensional reconstruction of each patient’s brain was computed using FreeSurfer (http://surfer.nmr.mgh.harvard.edu). Subdural electrodes were localized to neocortical regions in Freesurfer. MRI scans were visualized using Mango (Multi-image Analysis GUI, Research Imaging Institute, UTHSCSA). Hippocampal depth electrode location was confirmed by expert review (AL, DF).

**Figure 3:**
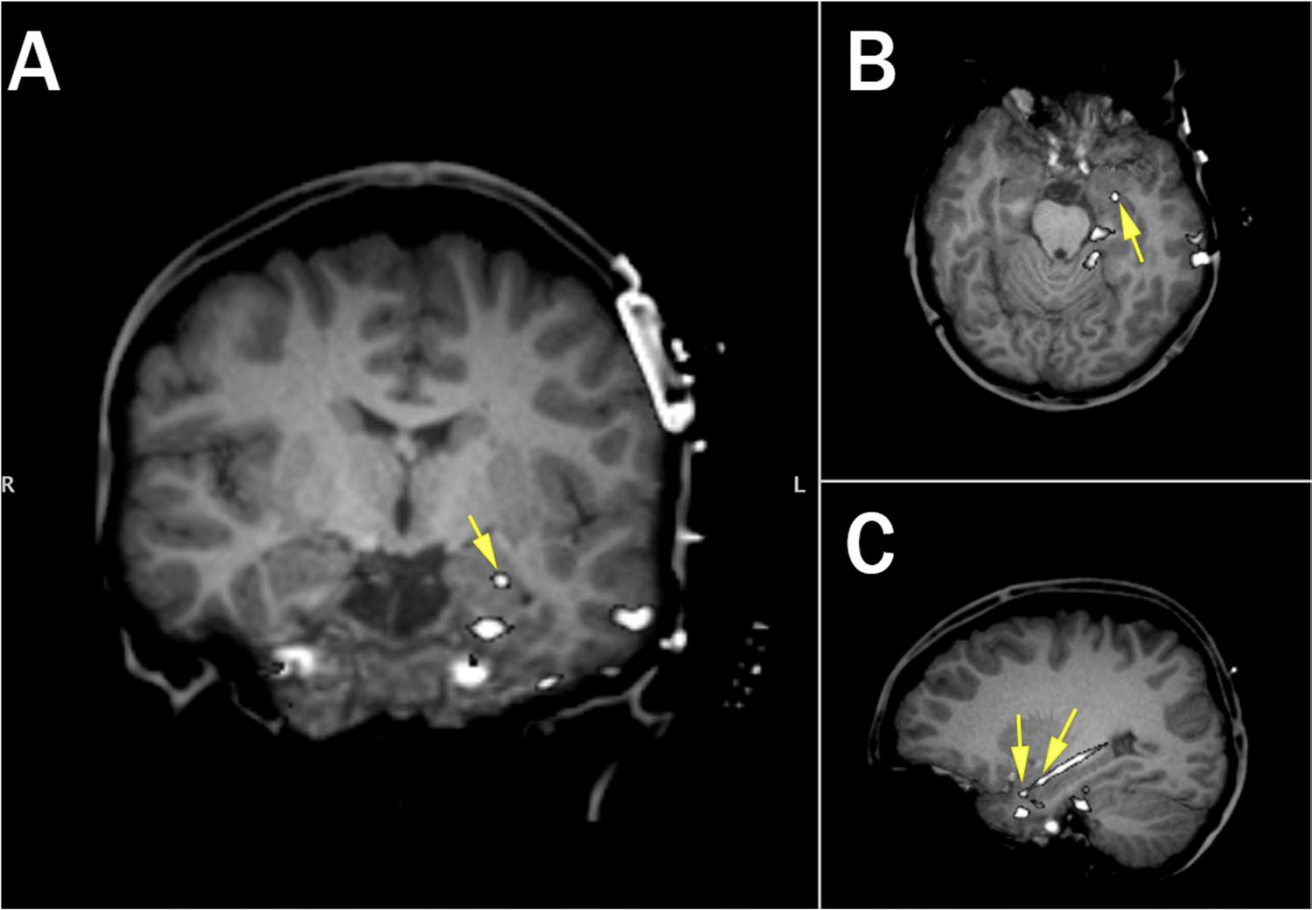
Electrode localization for RNS System patient (R1). Location of selected electrodes (Left Hipp 1–2) indicated by yellow arrows (**A**: Coronal, **B**: Axial, **C**: Sagittal; Coronal and Axial Views show only LH2).

**Figure 4:**
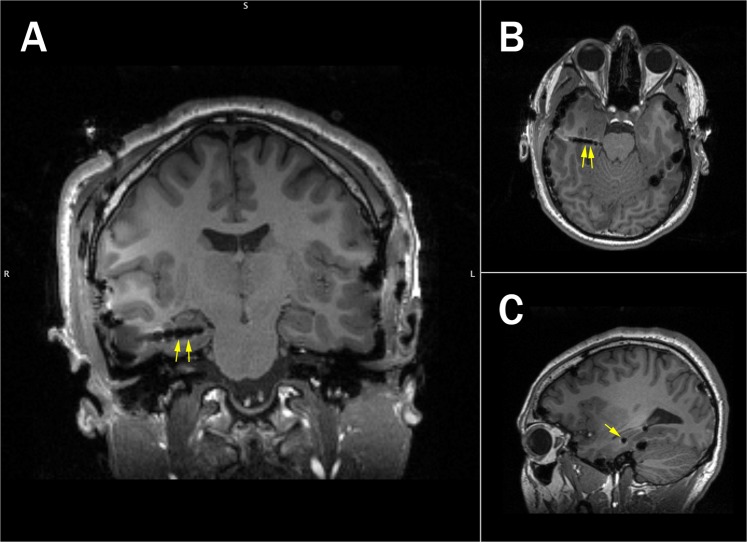
Electrode localization for an example surgical patient (S2). Location of selected electrodes is indicated with the yellow arrows (Right Anterior Temporal 03/04, **A**: Coronal, **B**: Axial, **C**: Sagittal).

Localization of the RNS System lead electrodes was performed by using linear co-registration of the pre-operative, skull-stripped MRI with the post-operative high-resolution CT (using BioimageSuite v3.0, Figs 3 and 4). Anatomical location of each contact was determined by visual inspection of the coregistered image (DF, AL).

Our main objective was to compare the hippocampal gamma response during associative learning in the RNS System to the gold standard of conventional iEEG recordings. Therefore, our primary criteria for selecting electrode pairs for analysis (due to the necessity of analysis in bipolar montage, explained under data analysis and statistics) in conventional iEEG was their shared location in hippocampus, as determined by coregistration of the electrodes to the pre-surgical MRI Brain within MNI space, and later confirmed by expert review. When there was more than one electrode pair in an individual patient which were located in hippocampus, we selected the pair with the lesser density of interictal discharges or line noise. Of note, most of the electrode pairs which were selected in the surgical iEEG patients were located in anterior hippocampus. This may be due to the larger volume of the hippocampal head, which can potentially accommodate two electrodes. As the the face-profession task has demonstrated a robust increase in fMRI BOLD signal in bilateral hippocampi^13^, both left and right hippocampal electrode pairs were eligible. To facilitate comparison with the RNS System, we selected the most anteriorly oriented electrode pairs with the best signal quality.

One electrode pair was selected per participant based on the anatomical localization. For surgical patients, electrode selection is provided in Supplementary Table S2.

### Data Analysis and Statistics

Hippocampal electrode pairs were selected according to electrode localization procedures described above, with expert validation by two independent reviewers (AL, DF). The RNS System records local field potentials (LFPs) in 4 channels, with each channel recording in a bipolar montage between 2 adjacent recording electrodes. Bipolar recording is a fixed feature of the RNS System, and cannot be re-referenced to other montages. Given these technical constraints, and the desire to standardize analytic techniques between RNS System and surgical iEEG, the surgical iEEG was re-referenced to a bipolar montage between selected electrode pairs to facilitate comparison in the primary analysis.

All candidate electrodes were inspected for signal quality by plotting the raw voltage tracings by trial. Electrodes were discarded based on high 60 Hz noise (likely due to poor contact impedance) and/or excessive interictal activity. Standard artifact rejection techniques, such as notch filtering for line noise and harmonics (60, 120, 180 Hz), detrending, and baseline correction were applied. In addition, EEG artifacts were removed by omitting trials where the raw signal exceeded 5 SD above the mean (across all trials per subject), as well as trials with excessive interictal epileptiform activity. For RNS System data, an additional data blanking routine was used to zero-out telemetry marker artifacts (“triggers”) delivered at stimulus onset (see Fig. 2).

Finally, visual inspection of individual trials was used to exclude trials with excessive non-physiological noise. Importantly, during signal analysis, we found that we discarded less iEEG data due to environmental noise or epileptiform activity in our RNS System patients compared to our surgical patients. Overall, approximately 20% (94/481) of trials were rejected from the surgical iEEG data, whereas only 5% (29/558) of the trials were rejected from the RNS System iEEG data. A summary of the trial rejection for each subject is in Table 1 (last column).

Analysis focused on identifying differences in the spectral-temporal features between successful and failed encoding trials. All trials from the selected electrodes were pooled for subsequent analyses. We performed time-frequency analyses on discrete task windows, including 1 s before and 2 s during encoding. EEG recordings were separated into trials of subsequently recalled and forgotten face-profession pairs. For each trial, gamma power was extracted using 8 semi-logarithmically spaced constant-Q Morlet wavelet filters (center frequencies between 62–96 Hz, 6 cycles), the amplitude squared, and normalized to the pre-stimulus baseline window (−0.5 to −0.05 s). The time-course of gamma power was also assessed by averaging the output of each wavelet filter across time. Differences between conditions (successful vs. failed encoding) was assessed using a non-parametric permutation test and controlled for multiple comparisons using a cluster-based correction^17^. Significance was determined at a 5% alpha level. A similar analysis was performed to estimate theta power, however, with slightly longer number of cycles to increase the frequency resolution at this lower frequency range (5 logarithmically spaced, 6–12 Hz, 8 cycles).

### Ethical Approval and Informed Consent

Research with RNS System and surgical epilepsy patients was performed in accordance with protocol approved by the New York University School of Medicine Institutional Review Board (NYUMC). Methods were carried out in accordance with the relevant guidelines and regulations, and with written patient consent. NeuroPace and RNS are registered trademarks of NeuroPace, Inc.

## Results and Discussion

Performance on the face-profession association task for the surgical patients varied (range 40.4–79.2% correct, mean 51.2 +/− 15.9 SD correct) as did performance by the RNS System patients (range 19.4–65.4% correct, mean 47.5 +/− 15.2 SD correct Table 1). During behavioral testing with our RNS System patients, we observed that our three RNS System patients were generally able to engage in memory testing for longer periods of time (as demonstrated by the larger block sizes, Table 1) compared to our surgical patients. Although responsive neurostimulation was temporarily disabled, there were no seizures or other complications during testing.

**Figure 5:**
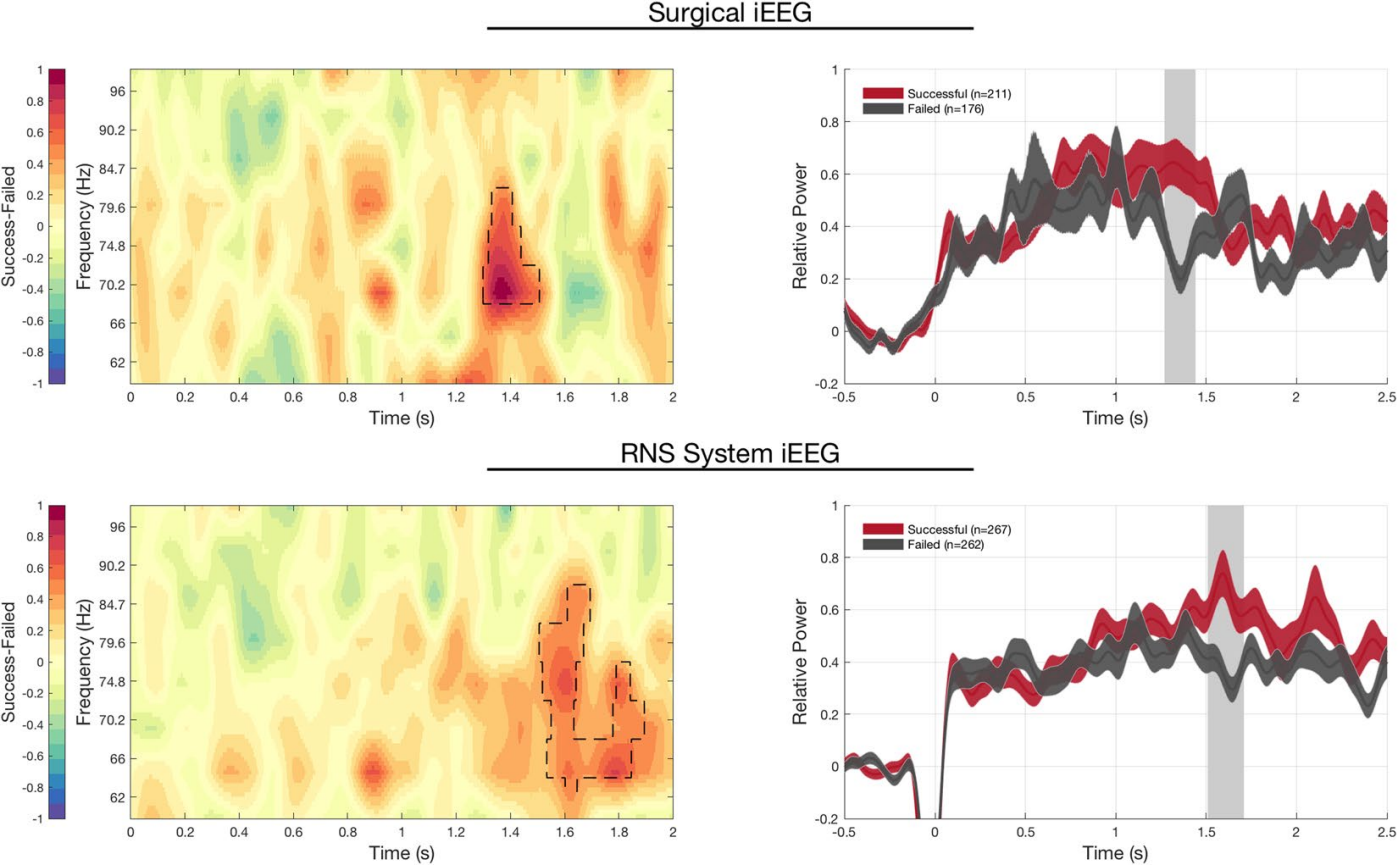
Hippocampal signature of successful versus failed encoding in RNS System and Surgical patients. Successful encoding shows increased and sustained gamma power in the hippocampus, compared to failed encoding in both RNS System iEEG and surgical iEEG recordings. LEFT. Time-frequency plot show increases in gamma power over similar frequency regions and timescales in successful versus failed encoding trials in both RNS System (top left) and surgical patients (bottom left). RIGHT: Gamma power in hippocampal lead electrodes exhibits a similar time course, with increased gamma power occurring 1.25–1.6 s from stimulus onset in both RNS System (top right) and surgical patients (bottom right; mean +/− SEM; dashed areas in spectrogram and shaded grey bar in the time-course indicates significant differences, p < 0.05 cluster-based permutation test. The drop in in power from −0.1–0.05 s in RNS System data is due to data blanking due to trigger artifact, see Methods).

Our main finding was hippocampal gamma (62–96 Hz) was greater from 1.3–1.5 seconds after stimulus presentation during encoding of face-profession pairs later successfully remembered compared to those later forgotten in our surgical epilepsy patients (Fig. 5, p < 0.05 cluster corrected permutation test, with 211 successful and 176 failed trials). Notably, there was substantial overlap in the frequency range of these gamma power increases across subjects (e.g. 70–85 Hz, Fig. 5 Left). Importantly, the hippocampal physiology supportive of successful encoding in our RNS System patients mirrored the physiological changes observed in our surgical patients **(**Fig. 5), with increased gamma activity 1.4–1.6 seconds after stimulus onset distinguishing successful versus failed encoding trials (Fig. 5, right, p < 0.05, cluster corrected permutation test, 267 successful, 262 failed trials). This result was also seen when the data were averaged across subjects, using the Fisher’s combined probability test (Supplementary Figure S2).

Furthermore, in both successful and failed encoding trials, there is a gradual increase in gamma seen during the early part of the encoding phase (prior to 1.3 sec). However, during the 1.3–1.6 second window, there is a sustained increase in gamma characteristic of successful, but not failed trials. A direct comparison between successful and failed encoding trials is provided in Supplementary Figure S1.

We also observed a simultaneous decrease in theta band power for surgical iEEG patients between 1.4–1.6 seconds, but not in our RNS System iEEG patients (Supplementary Figure 4 and 5). The discrepancy in our surgical iEEG and RNS iEEG data may due to differences in acute versus chronic iEEG recordings, attenuation of low frequency activity outside of the RNS System bandpass filter, or task design. Regarding the latter, our task was not designed to elicit a robust increase in theta activity, as seen in navigation studies utilizing intracranial EEG^12,18^. Of note, a recently published paper using RNS ECoG spectral features as biomarkers of antiepileptic drug response found a significant decrease in the lower frequency bands, including delta (0–4 Hz) and theta (4–8 Hz) activity, correlated with clinically beneficial antiepileptic drug initiation trials^19^.

Our finding of increased gamma activity during successful encoding resembles findings which have been previously described during word list learning and word pair tasks^8,20^. While the exact origin of the LFP is still debated^21–23^, gamma activity is commonly thought to arise from local neuronal activity (e.g. post-synaptic potentials and/or neural spiking)^24^.

By accessing chronic invasive EEG in the ambulatory setting, patient participation in cognitive tasks may be improved. Furthermore, the RNS System offers a unique opportunity to study memory dynamics over prolonged time scales. Previously, the RNS System iEEG recording has been used to investigate longitudinal neural dynamics using passive recordings. Evoked responses to phonetic features of speech perception and production have been demonstrated to be stable over 1.5 years^25^, and circadian and ultradian patterns of long term seizure dynamics have been revealed^26–28^. Given this demonstration of its ability to probe hippocampal dynamics, we suggest that the RNS System may become an important platform to investigate the cognitive neuroscience of memory.

The major limitation of the RNS System as a research tool is restricted electrode coverage based on the patient’s clinical indication. While lead electrodes are placed on or in epileptogenic tissue, the decision to avoid surgical resection suggests that the tissue is functional. Another consideration is that the RNS System only permits recording in a bipolar montage, which may exclude physiological signals occurring over broader regions of hippocampus. Finally, filter characteristics (i.e. high-pass filter at 4 Hz and low-pass filter at 120 Hz) attenuate functional activity known to fall outside the RNS System filter (further technical differences discussed in Supplementary Table S1). Furthermore, signals outside the bandpass frequency range are not directly comparable with those from other systems.

In summary, we found that increased hippocampal gamma activity distinguishes between successful and failed encoding trials during an associative learning paradigm in 5 epilepsy patients implanted acutely for surgical evaluation, as well as in 3 patients implanted chronically with the RNS System. Both successful and failed trials were characterized by early rises in gamma activity, however remembered trials demonstrated sustained increases in gamma activity between 1.3–1.6 seconds, whereas failed trials did not. Our findings replicate and extend prior work investigating the hippocampal subsequent memory effect, which has used verbal list learning and word-pair associative learning tasks.

Furthermore, we demonstrate a novel methodology of interfacing with the RNS System for task-based memory testing, and validate this method for making meaningful measurements of hippocampal dynamics. The technique incorporates event markers into the live streaming iEEG signal from the patient’s RNS System, allowing for later task-based iEEG analysis, without altering the clinical device. While the recording characteristics are more limited in the RNS System compared to traditional iEEG due to montage, amplifier and storage constraints, we have demonstrated that the RNS System EcoG has similar sensitivity for gamma band changes during a memory task compared to conventional iEEG. Of equal importance, we have demonstrated a method of adapting the task design for concurrent RNS System recording. While this study was performed in only a small number of patients, we have found that RNS System patients are able to engage in the memory task for longer sessions compared to our surgical patients (Table 1). Testing sessions in our RNS System patients were performed over the span of several months, which was not possible for the surgical patients.

Patients with chronically implanted brain-responsive neurostimulation devices (RNS System) present an opportunity to perform memory and other cognitive testing under more controlled conditions and across longer timescales compared to those typically used with conventional iEEG. Because the neurostimulator is chronically implanted and battery powered, investigations of neurophysiology during real-world activities are now possible^12^. Finally, the RNS System offers the potential of memory investigations conducted across more behaviorally relevant timescales (hours to days to months to years), compared to those typically used in research (seconds to minutes).

## Supplementary information

Supplementary Material

## Data Availability

Data is available upon reasonable request.
