# Supplementary material for: Hippocampal gamma predicts associative memory performance as measured by acute and chronic intracranial EEG
Source: Sci Rep. 2019 Jan 24;9:593. doi: 10.1038/s41598-018-37561-z (PMC6345863; doi:10.1038/s41598-018-37561-z)
Supplement: Supplementary file 1 — Supplementary Material [file 41598_2018_37561_MOESM1_ESM.docx]

**Supplementary Material**

**Hippocampal gamma predicts associative memory performance as measured by acute and chronic intracranial EEG**

Simon Henin*, Anita Shankar*, Nicholas Hasulak, Daniel Friedman, Patricia Dugan, Lucia Melloni, Adeen Flinker, Cansu Sarac, May Fang, Werner Doyle, Thomas Tcheng, Orrin Devinsky, Lila Davachi, Anli Liu

*denotes equal contribution

***Patients implanted with the RNS System.*** The three RNS System patients described below were implanted for on-label RNS System indication for use. Cognitive testing related to this study was performed at least 6 months after implantation.

**Subject R1** was a 21-year-old right handed man with a history of refractory left temporal lobe epilepsy due to focal cortical dysplasia in the medial bank of the left perirhinal cortex. His seizures began at the age of 13, with an aura of a “pleasant sensation,” a vague feeling of familiarity and “needing to figure something out, how to merge something together.” His seizures would progress to unresponsiveness and staring, and could progress to cyanosis. His brain MRI demonstrated a cortical abnormality involving the left perirhinal cortex, with brain PET hypometabolism involving the left lateral and basal temporal cortex. His intracarotid amobarbital (Wada) test demonstrated left hemispheric language dominance, with bilaterally supported memory function (Left 11/12, 91.6%; Right 12/12 100%). Neuropsychological testing showed that his overall level of intellectual functioning was above average (Wechsler Adult Intelligence Scale, FSIQ=108). During intracranial EEG monitoring, his typical focal seizures arose from the left anterior and basal temporal neocortex. Bedside cortical mapping demonstrated overlap between eloquent (language) cortex and the identified seizure onset zone, necessitating a tailored left temporal neocortical resection with implantation of the RNS System, with a left hippocampal depth and left lateral temporal subdural electrodes (**Figure 1**). Pathology of the resected temporal neocortex demonstrated focal cortical dysplasia (Type IIa).

**Subject R2** was a 43-year old right-handed man with a history of febrile seizures and traumatic brain injury from a remote motor vehicle accident with refractory bilateral temporal lobe epilepsy. His seizures were characterized by confusional episodes associated with oral and manual automatisms, with rocking movements, sometimes progressing to generalized tonic clonic seizures. Video EEG monitoring demonstrated seizures with bilateral independent temporal onsets. His brain MRI showed evidence of bilateral hippocampal sclerosis. On formal neuropsychological testing, his general level of functioning was in the low average range (FSIQ=84). His Wada test indicated left hemispheric language dominance, and significant right hemispheric memory dysfunction (Left 10/12, 83.3%; Right 0/12 0%). During intracranial EEG monitoring, the patient’s typical clinical seizures and numerous subclinical seizures arose from both left and right mesial temporal lobes. Because of the bilateral seizure onsets, the patient underwent placement of an RNS System, with bilateral hippocampal depth electrodes placed from an occipital approach (**Figure 1**).

**Subject R3** was a 32-year-old right handed woman with a history of refractory bilateral temporal lobe epilepsy from presumed viral encephalitis. As a sequelae of the encephalitis, she had residual memory impairment and refractory focal onset seizures. Her seizures began with a sensation of dizziness, followed by leftward or rightward head turn, then progressed to bilateral tonic clonic activity. Her brain MRI demonstrated right mesial temporal sclerosis, and brain PET revealed left lateral temporal hypometabolism. Wada testing revealed left hemispheric language dominance and bilaterally supported memory function (Left 11/12, 91.6%; Right 9/12, 75%). Formal neuropsychological testing showed that her overall level of intellectual functioning was in the average range (FSIQ 96). Intracranial EEG monitoring demonstrated several typical seizures arising from the left basal/lateral temporal lobe, as well as right mesial basal temporal lobe with rapid spread to the left temporal lobe. Because of the bilateral temporal lobe seizure onsets, she underwent placement of an RNS System, with bilateral hippocampal depth electrodes placed from an occipital approach (**Figure 1**).

***Surgical (S) Patients.***

**Subject S1** was a 45-year-old right handed woman with a history of refractory focal epilepsy from a severe traumatic brain injury, complicated by prolonged coma. She subsequently developed refractory seizures which were characterized by deja vu, followed by epigastric rising sensation and feeling of warmth enveloping her body, which could progress to confusion and face and hand automatisms lasting for one to two minutes. Her brain MRI demonstrated left hippocampal atrophy with signal abnormality suggestive of left mesial temporal sclerosis, mild right hippocampal atrophy, and encephalomalacia in the posterior left frontal lobe. Her Wada test demonstrated left sided language lateralization, and right hemisphere memory dysfunction (Left 11/12, 91.7%, Right -1.5/12, -12.5%). In formal neuropsychological functioning, her overall level of intellectual functioning was average (FSIQ 99). From intracranial monitoring, the patient was found to have seizures arising predominantly from the right mesial temporal lobe, and subsequently underwent a right anterior temporal lobectomy.

**Subject S2** was an 18-year-old right handed man with a history of refractory focal epilepsy for 3 years with unknown etiology. His seizures were characterized by (1) sudden behavioral arrest, followed by secondary generalized tonic clonic seizure, or (2) repetitive speech, alteration of awareness, and complex behaviors (hugging). His brain MRI showed possible FLAIR abnormalities in the left amygdala and left hippocampus. Brain PET MRI demonstrated mild bilateral hippocampal, medial temporal, and left lateral temporal FDG hypometabolism. His Wada testing demonstrated left language lateralization and bilaterally supported memory function (Left 12/12 100%, Right 12/12 100%). Formal neuropsychological testing demonstrated general intellectual functioning that was in the average range (FSIQ=91). During intracranial EEG monitoring, the patient was found to have multifocal hyperexcitability, more prominent in the left hemisphere, and focal epilepsy arising from the left hemisphere. Because of the multifocal nature of epileptogenic region, the patient was deemed to be a candidate for future implantation of the RNS System, and no surgical resection was performed.

**Subject S3** was a 37-year-old right handed male with a history of refractory seizures since 2005 from an unclear etiology. His seizures were characterized by deja vu, a rising epigastric sensation, then a strange smell, which could progress to shortness of breath and diaphoresis, and alteration of awareness and or to generalized tonic clonic seizures. His MRI Brain showed subtle FLAIR abnormalities and volume loss in his left hippocampus, as well as subtle right hippocampal volume loss. His brain MRI demonstrated subtle right anteromedial FDG hypometabolism. His video EEG demonstrated several (4) subclinical seizures arising from the left temporal region, with right greater than left temporal sharp and spike-wave discharges and bitemporal slowing. His Wada testing demonstrated left hemispheric language dominance and bilaterally supported memory function (Left 11/12 91.6%. Right 9/12 75%). His formal neuropsychological testing demonstrated that his general level of intellectual functioning was in the average range (TOPF, SS=95). During intracranial EEG monitoring, the patient was found to have frequent interictal discharges seen in the bilateral mesial temporal regions, and a single habitual seizure arising from the right temporal lobe. Thus, the patient was deemed to be a future candidate for the RNS System, and no surgical resection was performed.

**Subject S4** was a 20-year-old right handed woman with refractory right temporal lobe epilepsy. Her seizures were characterized by seizures characterized by leftward head turn progressing to secondary generalized tonic clonic seizure, occurring predominantly from nocturnal sleep. Her video EEG studies demonstrated subclinical and clinical seizures arising from the right temporal region, as well as right temporal epileptiform discharges. PET MRI Brain demonstrated right greater than left posterior cerebral hemisphere hypometabolism, and subtly decreased FDG uptake involving basal and lateral portions of the right temporal lobe. Her Wada testing demonstrated left hemispheric language dominance and bilaterally supported memory function (Left 12/12 100%, Right 12/12 100%). In formal neuropsychological testing, her general intellectual functioning was in the low average range (FSIQ 80). During intracranial EEG monitoring, the patient was found to have numerous clinical and subclinical seizures arising from the right temporal and parietal neocortical region, and subsequently underwent a right posterior temporal, inferior parietal cortical resection.

**Subject S5** was a 28-year-old right handed man with medication-resistant focal epilepsy of unclear etiology for two years. His seizures were characterized by onset of an intense sound, a sensation of slowed time, and intense panic, which occasionally progressed to alteration of awareness, and rarely to bilateral tonic clonic seizures. His ambulatory and video EEG studies demonstrated several typical seizures arising from the right anterior temporal lobe. PET MRI Brain showed right hippocampal atrophy but no focus of hypometabolism. In formal neuropsychological testing, general intellectual functioning was in the superior range (FSIQ=134). During intracranial monitoring, the patient was found to have multifocal interictal discharges arising from the right hemisphere, and numerous clinical seizures arising from the right anterior, mid, and mesial temporal regions. He subsequently underwent a right anterior temporal lobectomy.

**Supplementary Table 1. Comparison of RNS System and Conventional Recording Characteristics**

| **Feature** | **RNS System Characteristics  (NeuroPace, Inc.)** | **Intracranial EEG Amplifier Characteristics (Nicolet LTM, C64)** |
| --- | --- | --- |
| Electrodes | 4 electrodes per lead (depth or strip)  Depth: 1.27 mm Ø x 2.00 mm, 0.08 cm^2^ surface area  Strip: 3.175 mm Ø, 0.08 cm^2^ surface area  Electrode Spacing: 10 mm | 6-12 electrodes per lead (depths)  0.86 mm Ø  Electrode Spacing: 10 mm |
| ECoG Channels | 4 differential channels, 2 electrodes per amplifier user-selectable sensing montage | 64 monopolar channels per jackbox |
| Dynamic Range | 10-bit A/D conversion | 16-bit |
| Gain Levels | 4 gain levels (low, med-low, med-high, high) | N/A |
| Resolution | 0.8, 1.2, 2.0, 3.8 μV per bit | 0.153 μV |
| Sampling Rate | 250 samples/sec | 512 samples/sec |
| Low-pass Filter | -3dB at 30, 60, 90, 120 Hz (default = 90 Hz) | -3dB at 200 Hz |
| High-pass Filter | -3dB at 4, 8, 12 Hz (default = 4 Hz) | -3dB at 0.16Hz |
| ECoG Storage | 30.5 channel*minutes total storage  1 to 4 channels selectable  30, 60, 90, 180, 240 seconds duration 2/3 of ECoG record pre-trigger, 1/3 post-trigger 1-61 maximum ECoG records stored  (e.g. 30 4-channel 30-sec ECoG records)  Older ECoG records are overwritten unless reserved | Unlimited Disk Storage |
| ECoG Storage Triggers | Magnet, Responsive Therapy, Pattern A or B (Programmed) Detection, Long- Episode, Saturation, Noise, Scheduled | 12 non-isolated DC inputs (± 5V, BW = 100Hz) |

| 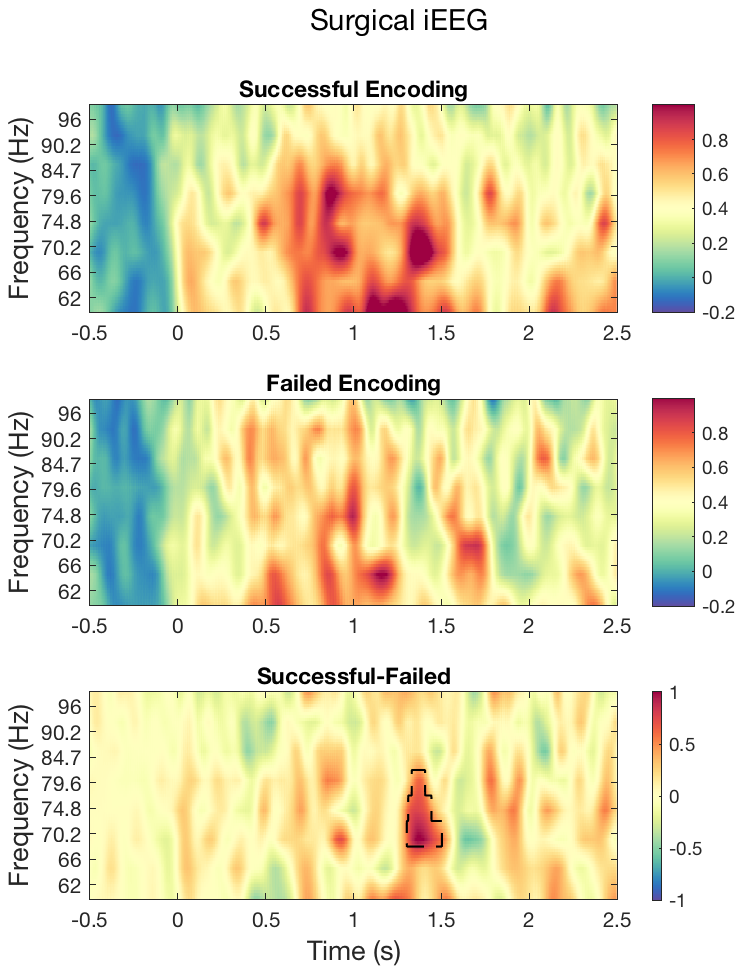 | 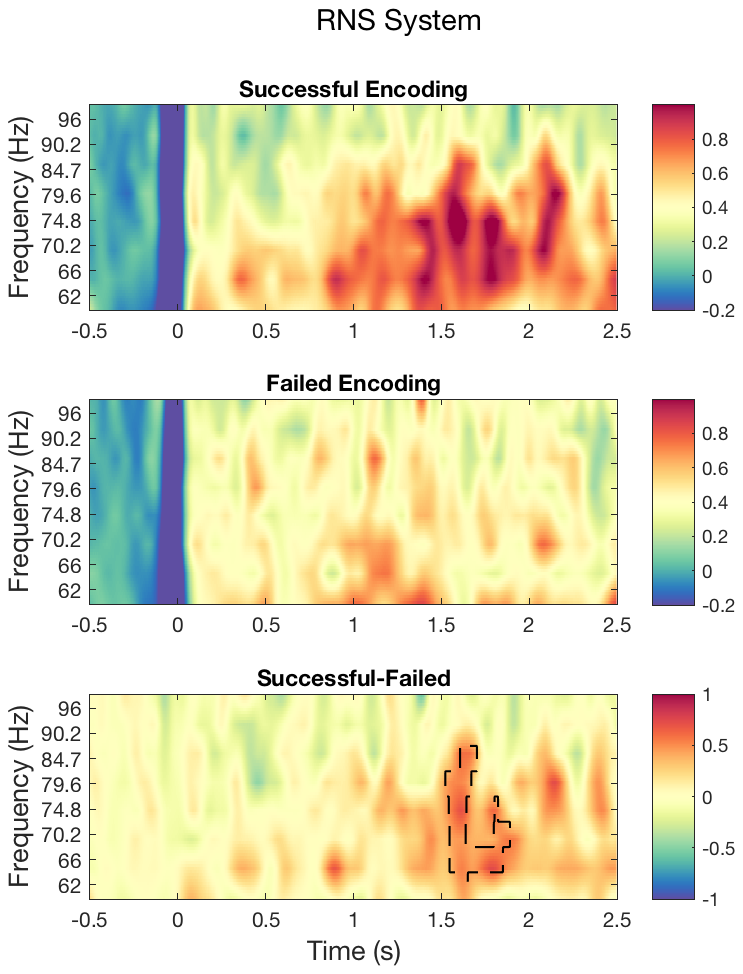 |
| --- | --- |

**Supplementary Figure S1. Spectro-temporal Differences between Successful vs. Failed Encoding States in the Hippocampus** Spectro-temporal changes in gamma power compared between successful and failed encoding trials in Surgical iEEG patients (Left) and RNS System patients (Right). In successful trials, there is a sustained increase in gamma activity between 0.5-2 seconds after presentation of the paired stimulus, whereas a less robust gamma response is seen during the time window during failed encoding trials, in both surgical and RNS patients. Significant differences between Successful (Top row) and Failed (Middle row) trials are shown as dashed outlines in the bottom panel (cluster-based permutation test). The dark band in RNS System patients (Right) just before time = 0, is caused by signal dropout during trigger markings.

**Reliability of responses across subjects as tested using the Fisher’s combined probability test.**

The reliability of the spectral changes across subjects was assessed by combining the p-values of individual subjects using Fisher’s combined probability test at each time point. Fisher’s combined probability test evaluates the assumption that all subjects’ null hypotheses are true, i.e. whether there is a consistent null effect across subjects, using the χ^2^ statistic. For each subject, p-values were computed independently for the time-frequency and time-course representations, using the same permutation procedure described in the Methods (**Data Analysis and Statistics**). P-values were then combined for each time-frequency bin (or time-point) using Fisher’s test, and FDR-corrected for multiple comparisons at q=0.05 and effects had to remain stable for a minimum of 10-ms to be considered significant. The results are shown in Supplementary Figure S2.

| Surgical iEEG | RNS System iEEG |
| --- | --- |
| 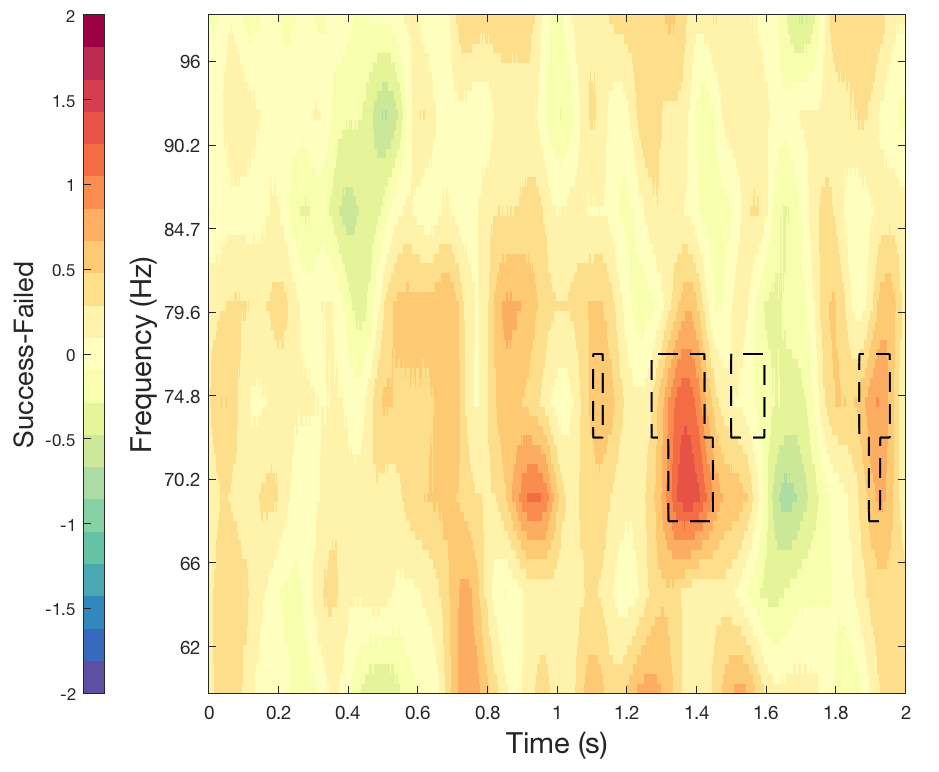 | 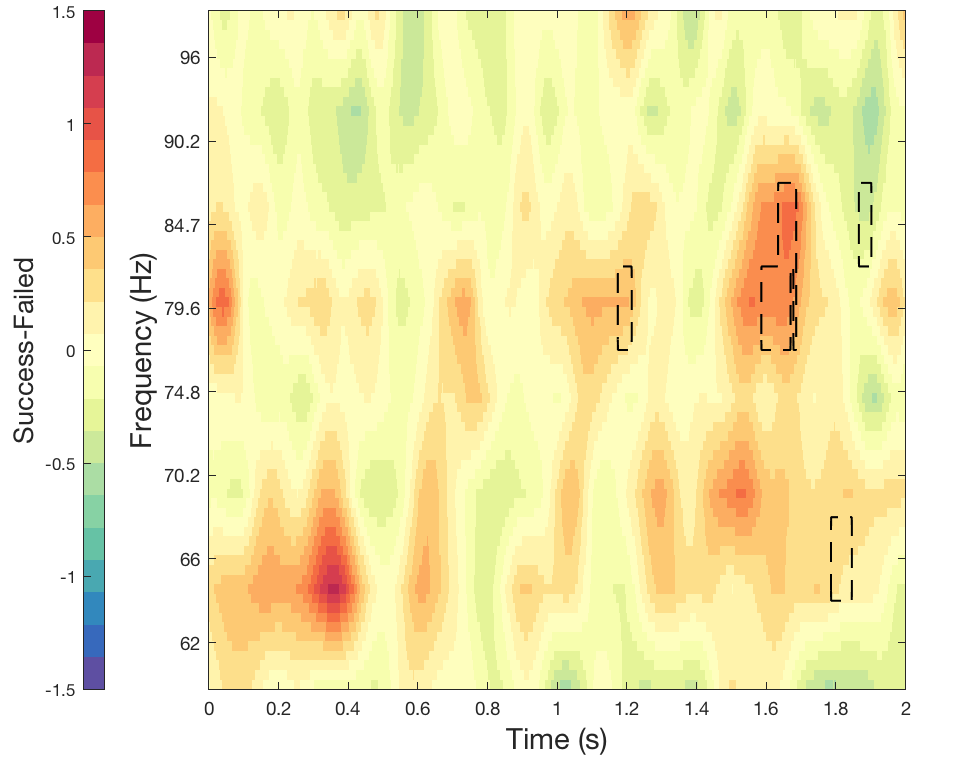 |

**Supplementary Figure S2. Single subject analysis of gamma power changes in Surgical iEEG patients** (**Left) and RNS System iEEG patients (Right).** Spectrogram of spectral power differences between successful versus failed encoding trials averaged across patients. Significant changes are outlined by dashed line.

**
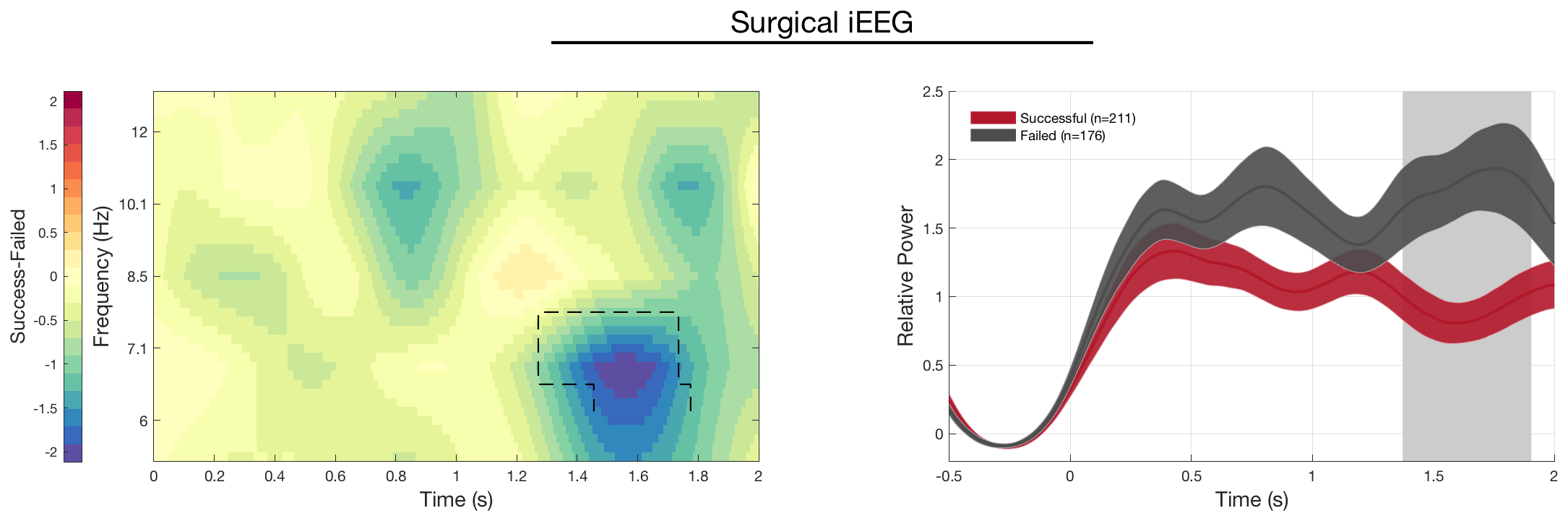
**

**Supplementary Figure S3. Analysis of Theta Band Changes in Surgical iEEG patients.** A significant reduction in theta power is observed between successful-failed encoding trials in time-frequency spectrogram (Left) as well as the overall power (Right) between 1.4-1.8 s post stimulus presentation, simultaneous to the increase in gamma band activity shown in Figure 5 and Supplementary Figure 1.


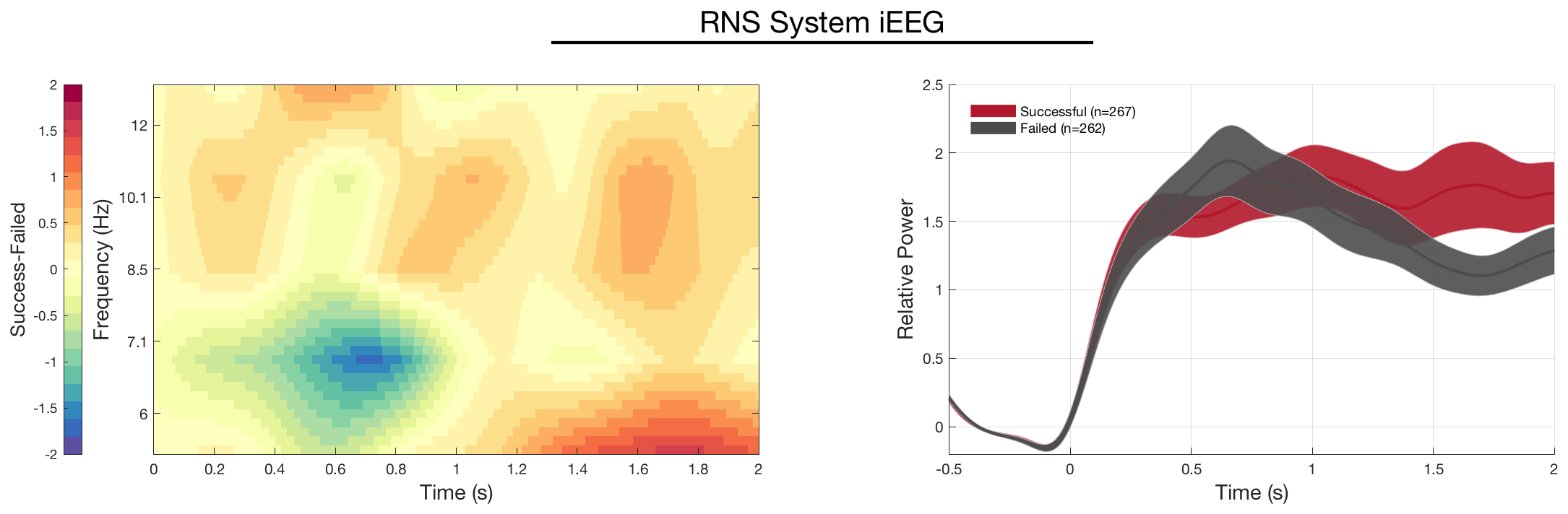


**Supplementary Figure S4. Analysis of Theta Band Changes in RNS System iEEG patients**. No significant changes in theta power were observed between successful vs. failed encoding trials in RNS System patients.
